# Evaluation of the Immunomodulatory Activities of the Probiotic Strain *Lactobacillus fermentum* UCO-979C

**DOI:** 10.3389/fimmu.2019.01376

**Published:** 2019-06-13

**Authors:** Valeria Garcia-Castillo, Ryoya Komatsu, Patricia Clua, Yuhki Indo, Michihiro Takagi, Susana Salva, Md. Aminul Islam, Susana Alvarez, Hideki Takahashi, Apolinaria Garcia-Cancino, Haruki Kitazawa, Julio Villena

**Affiliations:** ^1^Laboratory of Bacterial Pathogenicity, Faculty of Biological Sciences, University of Concepcion, Concepcion, Chile; ^2^Laboratory of Immunobiotechnology, Reference Centre for Lactobacilli (CERELA-CONICET), Tucuman, Argentina; ^3^Food and Feed Immunology Group, Laboratory of Animal Products Chemistry, Graduate School of Agricultural Science, Tohoku University, Sendai, Japan; ^4^Livestock Immunology Unit, International Education and Research Center for Food Agricultural Immunology (CFAI), Graduate School of Agricultural Science, Tohoku University, Sendai, Japan; ^5^Department of Medicine, Faculty of Veterinary Science, Bangladesh Agricultural University, Mymensingh, Bangladesh; ^6^Laboratory of Plant Pathology, Graduate School of Agricultural Science, Tohoku University, Sendai, Japan; ^7^Plant Immunology Unit, International Education and Research Center for Food Agricultural Immunology, Graduate School of Agricultural Science, Tohoku University, Sendai, Japan

**Keywords:** *Lactobacillus fermentum* UCO-979C, intestinal immunity, macrophages, PIE cells, immunobiotics

## Abstract

*Lactobacillus fermentum* UCO-979C, a strain isolated from a human stomach, was previously characterized by its potential probiotic properties. The UCO-979C strain displayed the ability to beneficially regulate the innate immune response triggered by *Helicobacter pylori* infection in human gastric epithelial cells. In this work, we conducted further *in vitro* studies in intestinal epithelial cells (IECs) and *in vivo* experiments in mice in order to characterize the potential immunomodulatory effects of *L. fermentum* UCO-979C on the intestinal mucosa. Results demonstrated that the UCO-979C strain is capable to differentially modulate the immune response of IECs triggered by Toll-like receptor 4 (TLR4) activation through the modulation of TLR negative regulators' expression. In addition, we demonstrated for the first time that *L. fermentum* UCO-979C is able to exert its immunomodulatory effect in the intestinal mucosa *in vivo*. The feeding of mice with *L. fermentum* UCO-979C significantly increased the production of intestinal IFN-γ, stimulated intestinal and peritoneal macrophages and increased the number of Peyer's patches CD4^+^ T cells. In addition, *L. fermentum* UCO-979C augmented intestinal IL-6, reduced the number of immature B220^+^CD24^high^ B cells from Peyer's patches, enhanced the number of mature B B220^+^CD24^low^ cells, and significantly increased intestinal IgA content. The results of this work revealed that *L. fermentum* UCO-979C has several characteristics making it an excellent candidate for the development of immunobiotic functional foods aimed to differentially regulate immune responses against gastric and intestinal pathogens.

## Introduction

It is widely recognized that commensal microorganisms are relevant for human and animals health, participating in several important biological functions including nutrients digestion, vitamins synthesis, and pathogens inhibition (1, 2). The importance of beneficial microorganisms of the microbiota in the maintenance of immune health was also demonstrated in a convincing way (3, 4). Several effective tools have already been developed in order to study and manipulate the microbiota, improve their beneficial properties for the host, and protect against immune-related diseases (5, 6). In this regard, the development of immunomodulatory probiotic (immunobiotic) interventions offers opportunities for the modulation of the mucosal immune system toward long lasting health (7).

The beneficial role of immunobiotic lactic acid bacteria (LAB) has been extensively reported, supporting their implementation to improve some immunological functions in the host (8–10). The beneficial effect of immunobiotics on the immune system occurs through direct and indirect interactions of bacteria with immune and non-immune cells (11–14), leading to cells' activation and proliferation, cytokines production, IgA secretion, antimicrobial peptides synthesis, and tight junctions improvement (2, 11–14). Several research works including recent transcriptomic analysis revealed that the immunomodulatory effect of immunobiotics is a strain-specific characteristic (2, 12, 15), and therefore, each individual strain has to be studied in detail in order to explore its immunomodulatory potential.

Immunobiotics has been proposed as an alternative to prevent bacterial and viral infections in gastrointestinal tract (10). Experimental models have demonstrated that immunobiotics can attenuate the severity of infections caused by several gastrointestinal pathogens including *Helicobacter pylori, Citrobacter rodentium, Listeria monocytogenes*, and *Salmonella typhimurium* (16). In this regard, it was reported that some *Lactobacillus* strains are able to increase the resistance against the gastric pathogen *H. pylori*. Among the mechanisms proposed to explain the beneficial effects of probiotic lactobacilli are the production of antimicrobial compounds, the inhibition of pathogen's adhesion and the modulation of the immune system (17–19). We previously isolated *Lactobacillus fermentum* UCO-979C from a human stomach sample and characterized its potential probiotic properties (20, 21). We observed that this strain is able to efficiently adhere to the gastric mucosa as demonstrated by *in vitro* experiments in human gastric adenocarcinoma cell-line (AGS cells) and *in vivo* studies in Mongolian gerbils (21). *L. fermentum* UCO-979C also showed the ability to strongly inhibit the adhesion, growth and urease activity of *H. pylori* (21, 22). Moreover, we recently reported that the UCO-979C strain beneficially modulates the innate immune response triggered by *H. pylori* infection in human gastric epithelial cells and macrophages (23). Our data showed reduced levels of the pro-inflammatory factors IL-8, TNF-α, IL-1β, IL-6, and MCP-1 expressions in *L. fermentum* UCO-979C-treated AGS cells when compared to untreated infected controls. In addition, improved production of the regulatory cytokine TGF-β in response to *H. pylori* infection was detected in *L. fermentum* UCO-979C-treated AGS cells (23). Interestingly, *L. fermentum* UCO-979C was also capable of reducing the production of TNF-α and improving IFN-γ and IL-10 levels in macrophages challenged with *H. pylori* (23).

Taking into consideration the effect of *L. fermentum* UCO-979C on the gastric immune response against *H. pylori* infection, we wonder whether this probiotic *Lactobacillus* strain is also able to modulate immune responses in the intestinal tract. Therefore, we conducted *in vitr*o studies in intestinal epithelial cells (IECs) and *in vivo* experiments in mice in order to characterize the immunomodulatory effects of *L. fermentum* UCO-979C on the intestinal mucosa.

## Materials and Methods

### Microorganisms

*Lactobacillus fermentum* UCO-979C was obtained from the Bacterial Pathogenicity Laboratory culture collection at University of Concepción (Concepcion, Chile) (20, 21). *L. fermentum* CRL973 was obtained from the CERELA culture collection (Tucuman, Argentina). Lactobacilli strains were activated from frozen stock and cultured in Mann-Rogosa Sharpe Agar (MRS Difco) at 37°C. After 24 h incubation, a single colony was transferred to MRS broth (MRS Difco) and cultured at 37°C for 24 h. Then, bacterial cells were washed three times with phosphate buffered saline (PBS) and adjusted to appropriate concentrations for the *in vitro* and *in vivo* experiments.

Enterotoxigenic *Escherichia coli* (ETEC) strain 987P (O9: H-: 987 pilus+: heat stable toxin+) was obtained from the National Institute of Animal Health (Tsukuba, Japan) (24–26). ETEC cells were grown in blood agar (5% sheep blood) for 24 h at 37°C and transferred to tryptic soy broth (TSB; Becton, Dickinson and Company, USA) and cultured 20 h at 37°C with shaking. After incubation, the subcultures of bacteria were centrifuged at 5,000 × g for 10 min at 4°C and washed with PBS (pH 7.2). Finally, ETEC cells were heat killed at 100°C for 15 min and then washed with PBS. Heat-stable ETEC pathogen associated molecular patterns (PAMPs) were suspended in DMEM for the experimental challenge.

*Sacharomyces boulardi* was obtained from commercial lyophilized preparation (Floratil—Argentina). The yeast suspensions were prepared in PBS and heat inactivated during 15 min in 100°C water bath and adjusted to 10^7^ cells/ml for *ex vivo* phagocytosis assay.

### Porcine Intestinal Epitheliocytes

Porcine intestinal epitheliocytes (PIE cells) are non-transformed intestinal cultured cells derived from intestinal epithelia isolated from an unsuckled neonatal swine. When PIE cells are cultured, they assumed a monolayer, cobblestone, and epithelial-like morphology, with close contact between cells (24). PIE cells were maintained in Dulbecco's modified Eagle's medium (DMEM) (Invitrogen Corporation, Carlsbad, CA, USA) supplemented with 10% fetal calf serum FCS (Hyclone, Logan, USA), 100 mg/ml penicillin, and 100 U/ml streptomycin at 37°C in an atmosphere of 5% CO_2_. PIE cells grow rapidly and are well adapted to culture conditions even without transformation or immortalization. However, the proliferative ability of PIE cells diminishes after 50 passages in culture. Therefore, we used PIE cells only between the 20th and 40th passages in these experiments (25, 26). Briefly, PIE cells were cultured in 250 ml flasks (1.0 ×10^6^ cells) for 5 days after reaching 80–90% confluence, changing culture media every 1–2 days, followed by subculturing in 24 well flasks for immunostimulation assays as described below.

### Immunomodulatory Assay in PIE Cells

Lactobacilli were re-suspended in DMEM (10% FCS), enumerated in a microscope using a Petroff-Hausser counting chamber, and stored at −80°C until use. PIE cells were plated at 3 ×10^4^ cells/well of a 12-well type I collagen-coated plates (Iwaki, Tokyo, Japan), and cultured for 3 days. After changing medium, lactobacilli (5 ×10^8^ cells/ml) were added followed by stirring in microplate mixer and co- cultured for 48 h at 37°C 5% CO_2_ atmosphere. Then, each well was washed vigorously with medium at least 3 times to eliminate bacteria. Gene expression of cytokines, chemokines, complement, and coagulation factors as well as negative regulators of TLR signaling were studied without any inflammatory challenge (basal levels) or after heat-stable ETEC PAMPs challenge (5 ×10^7^ cells/ml) for 12 h (25) by using RT-PCR as described below.

### Quantitative Expression Analysis by RT-PCR

Two-step real-time qPCR was performed to characterize the expression of selected genes in PIE cells. Total RNA was isolated from each PIE cell sample using TRIzol reagent (Invitrogen). RNA purity and concentration were assessed using NanoDrop^TM^ 1,000 Spectophotometer. All cDNAs were synthesized using the PrimeScript RT Reagent kit with the treatment of gDNA eraser (Takara—Bio, Japan) according to the manufacturer's recommendations. Real-time quantitative PCR (qRT-PCR) was carried out using a 7,300 real-time PCR system (Applied Biosystems, Warrington, UK) and the Platinum SYBR green qPCR SuperMix uracil-DNA glycosylase (UDG) with 6-carboxyl- X-rhodamine (ROX) (Invitrogen). The primers used in this study were described previously (25–27). The PCR cycling conditions were 2 min at 50°C, followed by 5 min at 95°C, and then 40 cycles of 15 s at 95°C, 30 s at 60°C, and 30 s at 72°C, followed by a dissociation stage of 15 s at 95°C, 1 min at 60°C, 15 s at 95°C and 15 s at 60°C. The reaction mixtures contained 2.5 μl of cDNA and 7.5 μl of master mix, which included the sense and antisense primers. According to the guidelines for minimum information for publication of qRT-PCR experiments, β-actin was used as a housekeeping gene, to normalize cDNA levels for differences in total cDNA levels in the samples, because of its high stability across porcine various tissues (28). The relative index of a cytokine mRNA in PIE cells stimulated with lactobacilli was calculated as follows: first, the average cytokine expression levels from at least three samples challenged with ETEC without prestimulation with lactobacilli were set to 100. Then, relative expressions of cytokines were calculated in the samples prestimulated with lactobacilli followed by ETEC challenge (25).

### Animals and Feeding Procedures

Female 6–8 weeks old Balb/c mice were obtained from the closed colony kept at CERELA (Tucuman, Argentina). They were housed in plastic cages with controlled room temperature (22 ± 2°C temperature, 55 ± 2% humidity) and were fed *ad libitum* conventional balanced diet. Animal welfare was ensured by researchers and special trained staff in animal care and handling at CERELA. Animal health and behavior were monitored twice a day. This study was carried out in strict accordance with the recommendations in the Guide for the Care and Use of Laboratory Animals of the Guidelines for Animal Experimentation of CERELA. The CERELA Institutional Animal Care and Use Committee prospectively approved this research under the protocol BIOT-CRL-17.

Mice were housed individually during the experiments and the assays for each parameter studied were performed in 5–6 mice per group. *L. fermentum* UCO-979C or CRL973 were administered to different groups of mice for 2 consecutive days at a dose of 10^8^ cells/mouse/day in the drinking water (4 ml per mice per day). Bacteria were prepared as described above, suspended in 5 ml of 10% skimmed milk and added to the drinking water.

In some experiments, 1 day after at the end of the lactobacilli treatments, mice were challenged with lipopolysaccharide (LPS) to induce inflammation. Mice received 8 mg/kg of LPS from *E. coli* O55:B5 by intraperitoneal injection.

### *Ex vivo* Phagocytosis Assay

Peritoneal macrophages were collected aseptically from mice as described previously (29, 30). Briefly, after exposing inner skin, cold PBS containing 10% fetal bovine serum was carefully injected into peritoneal cavity. The fluid was collected and macrophages were washed twice with PBS containing bovine serum albumin (BSA) and adjusted to a concentration of 1 ×10^6^ cells/ml. Phagocytosis was performed using a heat-killed *S. boulardi*. Mixtures of opsonized yeasts in mouse autologous serum (10%) were added to 0.2 ml of macrophage suspension. The mixture was incubated for 30 min at 37°C. The percentage of phagocytosis was expressed as the percentage of phagocytizing macrophages in 200 cells counted using an optical microscope (30, 31).

### Nitro Blue Tetrazolium Test (NBT)

The bactericidal activity (oxidative burst) of peritoneal macrophages was measured using the NBT reduction test (Sigma-Aldrich, St. Louis, MO, USA) as previously described (30). Briefly, 200 μl of peritoneal macrophages obtained as described above were incubated with 120 μl of NBT reagent and incubated first at 37°C for 10 min and then 10 min at room temperature. NBT was added to each sample and incubated at 37°C for 20 min. In the presence of oxidative metabolites, NBT (yellow) is reduced to formazan, which forms a blue precipitate. Smears were prepared and, after staining, the samples were examined under a light microscope for blue precipitates. Randomly, 100 cells were counted and the percentage of NBT positive (+) cells were determined (30, 31).

### Cytokine Concentrations

The concentration of cytokines was determined in blood and intestinal samples of UCO-979C-tretated and control mice. Blood samples were obtained through cardiac puncture at the end of each treatment and collected in heparinized tubes. Intestinal fluid samples were obtained as the method described previously (31). Briefly, the small intestine was flushed with 5 ml of PBS and the fluid was centrifuged (10,000 × g, 4°C for 10 min) to separate particulate material. The supernatant was kept frozen until use. Tumor necrosis factor α (TNF-α), interferon γ (IFN-γ), and interleukin 10 (IL-10) concentrations in serum and intestinal fluid, were measured with commercially available enzyme-linked immunosorbent assay (ELISA) kits following the manufacturer's recommendations (R&D Systems, MN, USA).

### Flow Cytometry

Peritoneal macrophages were collected as described above. Peyer's patches were collected and mechanically disaggregated. A single-cell suspension from the Peyer's patches of each mouse was obtained by gently passing the collected tissue through a tissue strainer with PBS with 2% FCS (FACS buffer). Cell suspensions were subjected to red blood cells lysis (Tris-ammonium chloride, BD PharMingen) flowed by counting on a hemacytometer. Viability of cells was assessed by trypan blue exclusion. Cell suspensions were pre-incubated with anti-mouse CD32/CD16 monoclonal antibody (Fc block) for 30 min at 4°C. Cells were incubated with the antibody mixes for 30 min at 4°C and washed with FACS buffer. The following antibodies from BD Biosciences were used: FITC-labeled anti-mouse MHC-II, FITC-labeled anti-mouse CD86, PE-labeled anti-mouse CD11b, PE-labeled anti-mouse F4/80, PE-labeled anti-mouse Ly6C, PE-labeled anti-mouse CD24, biotinylated anti-mouse B220, FITC-labeled anti-mouse CD3, PE-labeled anti-mouse CD8, and biotinylated anti-mouse CD4 antibodies. Streptavidin-PerCP was used as a second-step reagent. Flow cytometry was performed using a BD FACSCaliburTM flow cytometer (BD Biosciences) and data were analyzed using FlowJo software (TreeStar).

### Statistical Analysis

Experiments were performed in triplicate and results expressed as the mean ± SD. For the comparison of two groups, the Student's *t*-test was used. For the comparison of more than two groups, a one-way analysis of variance (ANOVA) was performed followed by and Tukey's test. In all cases, a level of significance of *p* < 0.05 was considered.

## Results

### *L. fermentum* UCO-979C Modifies Cytokine Profile in PIE Cells

**Figure 1:**
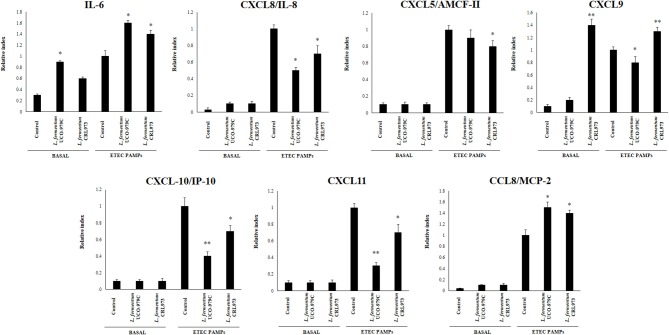
Effect of *Lactobacillus fermentum* UCO-979C and *L. fermentum* CRL973 on the expression of cytokines and chemokines in porcine intestinal epithelial (PIE) cells. PIE cells were pre-treated with UCO-979C or CRL973 strains for 48 h and then stimulated with heat-stable Enterotoxigenic *Escherichia coli* (ETEC) pathogen-associated molecular patterns (PAMPs). The expression of cytokines (IL-6) and chemokines (CXCL5, CXCL8, CXCL9, CXCL10, CXCL11, and CCL8) were studied at 48 h after lactobacilli stimulation (basal) or at 12 h after heat-stable ETEC PAMPs challenge. The results represent three independent experiments. Results are expressed as mean ± SD. Significantly different from control PIE cells at the same time point *(*P* < 0.05), **(*P* < 0.01).

**Figure 2:**
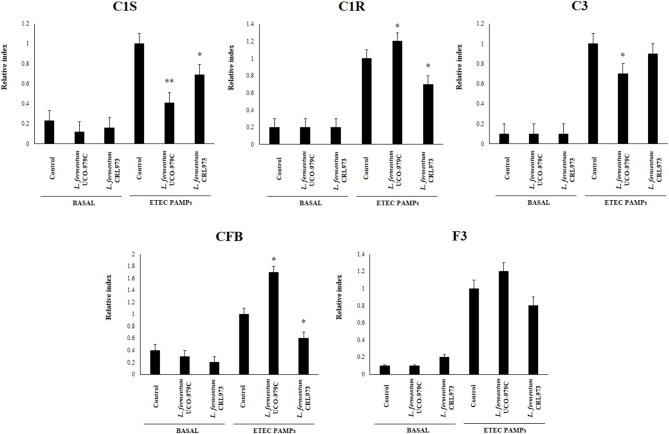
Effect of *Lactobacillus fermentum* UCO-979C and *L. fermentum* CRL973 on the expression of factors from the complement and coagulation systems in porcine intestinal epithelial (PIE) cells. PIE cells were pre-treated with UCO-979C or CRL973 strains for 48 h and then stimulated with heat-stable Enterotoxigenic *Escherichia coli* (ETEC) pathogen-associated molecular patterns (PAMPs). The expression of factors from the complement (C1S, C1R, C3, and CFB) and coagulation (F3) systems were studied at 48 h after lactobacilli stimulation (basal) or at 12 h after heat-stable ETEC PAMPs challenge. The results represent three independent experiments. Results are expressed as mean ± SD. Significantly different from control PIE cells at the same time point *(*P* < 0.05), **(*P* < 0.01).

We first evaluated whether *L. fermentum* UCO-979C was able to modify the cytokine expression profile of PIE cells by evaluating the mRNA levels of IL-6, CXCL8 (IL-8), CXCL5 (AMCF-II), CXCL9, CXCL10 (IP-10), CXCL11, and CCL8 (MCP1) as shown in Figure 1. Then, in order to evaluate whether the immunomodulatory effects of *L. fermentum* UCO-979C were a strain specific property, we performed comparative experiments with the strain of the same species *L. fermentum* CRL973. Stimulation of PIE cells with the UCO-979C or CRL973 strains increased the expression of IL-6 and CXCL9, respectively, while no differences were found between controls and lactobacilli-treated PIE cells when the other chemokines were analyzed. The modulation of cytokines and chemokines was also studied in the context of inflammation. For this purpose, PIE cells were treated with lactobacilli and then challenged with heat-stable ETEC PAMPs that are able to trigger Toll-like receptor 4 (TLR4) activation in this cell line (25, 26). Untreated PIE cells challenged with ETEC were used as controls. Heat-stable ETEC PAMPs significantly increased the expression of all the inflammatory cytokines and chemokines in all the experimental groups (Figure 1). However, the mRNA expression levels of IL-6 and CCL8 were significantly higher in lactobacilli-treated cells. In addition, expression of CXCL8, CXCL10, and CXCL11 were lower in lactobacilli-treated PIE cells than in controls. Interestingly, only *L. fermentum* CRL973 was able to reduce CXCL5 expression (Figure 1). *L. fermentum* CRL973 increased CXCL9 expression after ETEC challenge while the UCO-979C strain reduced the expression of this chemokine (Figure 1). Our previous immunotranscriptomics studies in PIE cells revealed that in addition to cytokines and chemokines, immunomodulatory probiotic strains are able also to modulate factors from the complement and coagulation systems (27). Therefore, we evaluated the expressions of C1S, C1R, C3, CFB, and F3 in PIE cells under inflammatory and non-inflammatory conditions (Figure 2). No significant expression differences were observed for these factors when control and lactobacilli-treated PIE cells were compared. Challenge with heat-stable ETEC PAMPs increased C1S, C1R, C3, CFB, and F3 expressions in all the experimental groups. *L. fermentum* UCO-979C-treated PIE cells had significantly lower levels of C1S and C3, and higher levels of C1R and CFB expression as compared to controls. While *L. fermentum* CRL973-treated PIE cells exhibited significantly lower levels of C1S, C1R, and CFB than controls (Figure 2). No significant differences were observed in F3 expression when control and lactobacilli-treated PIE cells were compared.

### *L. fermentum* UCO-979C Modifies Negative Regulators of TLR4 Signaling in PIE Cells

**Figure 3:**
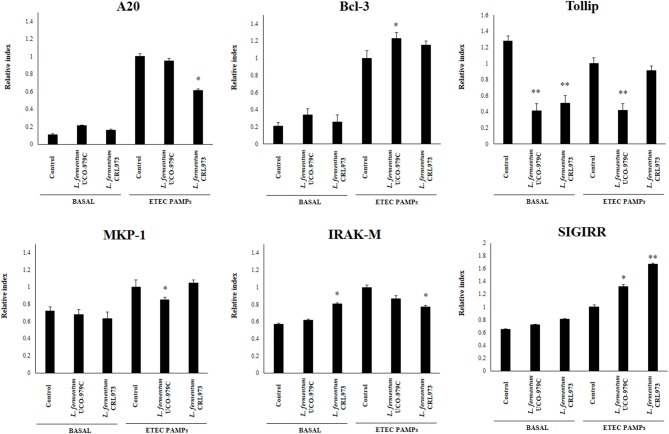
Effect of *Lactobacillus fermentum* UCO-979C and *L. fermentum* CRL973 on the expression of negative regulators of the Toll-like receptor (TLR) signaling pathway in porcine intestinal epithelial (PIE) cells. PIE cells were pre-treated with UCO-979C or CRL973 strains for 48 h and then stimulated with heat-stable Enterotoxigenic *Escherichia coli* (ETEC) pathogen-associated molecular patterns (PAMPs). The expression of negative regulators of the TLR signaling pathway were studied at 48 h after lactobacilli stimulation (basal) or at 12 h after heat-stable ETEC PAMPs challenge. The results represent three independent experiments. Results are expressed as mean ± SD. Significantly different from control PIE cells at the same time point *(*P* < 0.05), **(*P* < 0.01).

We next evaluated whether *L. fermentum* UCO-979C was able to modify the expression of negative regulators of TLR4 signaling in PIE cells (Figure 3). No significant differences were observed in the expression of A20, Bcl3, MKP-1, and SIGIRR when untreated control, and lactobacilli-treated PIE cells were compared. Both, UCO-979C and CRL973 strains were able to reduce the expression of Tollip in PIE cells, while *L. fermentum* CRL973 increased IRAK-M expression. Challenge with heat-stable ETEC PAMPs increased A20, Bcl3, MKP-1, IRAK-M, and SIGIRR in all the experimental groups. *L. fermentum* UCO-979C-treated PIE cells had significantly lower levels of MKP-1 and Tollip, and higher levels of Bcl3 than controls, while *L. fermentum* CRL973 showed significantly lower levels of A20 and IRAK-M expressions than controls (Figure 3). Both lactobacilli improved the expression of SIGIRR.

### *L. fermentum* UCO-979C Modulates Intestinal Immunity *in vivo*

**Figure 4:**
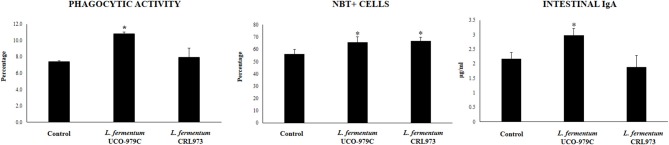
Effect of *Lactobacillus fermentum* UCO-979C and *L. fermentum* CRL973 on peritoneal macrophages activities and intestinal IgA production in adult immunocompetent mice. *L. fermentum* UCO-979C or CRL973 were administered to different groups of mice for 2 consecutive days at a dose of 10^8^ cells/mouse/day. Untreated mice were used as controls. One day after the last lactobacilli administration, phagocytic and bactericidal (oxidative burst) activities of peritoneal macrophages, and intestinal IgA concentrations were determined. The results represent three independent experiments. Results are expressed as mean ± SD. Significantly different from control mice *(*P* < 0.05).

**Figure 5:**
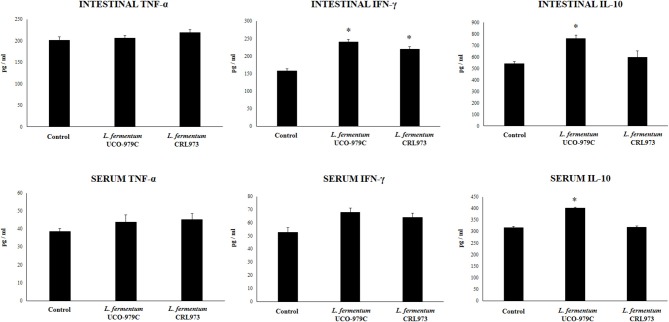
Effect of *Lactobacillus fermentum* UCO-979C and *L. fermentum* CRL973 on intestinal and serum cytokines of adult immunocompetent mice. *L. fermentum* UCO-979C or CRL973 were administered to different groups of mice for 2 consecutive days at a dose of 10^8^ cells/mouse/day. Untreated mice were used as controls. One day after the last lactobacilli administration, the concentrations of TNF-α, IFN-γ, and IL-10 in intestinal fluid and serum were determined. The results represent three independent experiments. Results are expressed as mean ± SD. Significantly different from control mice *(*P* < 0.05).

**Figure 6:**
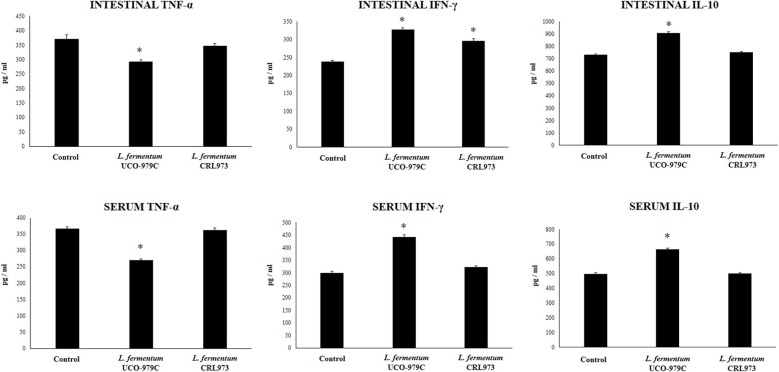
Effect of *Lactobacillus fermentum* UCO-979C and *L. fermentum* CRL973 on intestinal and serum cytokines of adult immunocompetent mice after lipopolysaccharide (LPS) challenge. *L. fermentum* UCO-979C or CRL973 were administered to different groups of mice for 2 consecutive days at a dose of 10^8^ cells/mouse/day. Untreated mice were used as controls. Lactobacilli-treated and control mice were challenged with LPS by intraperitoneal injection. One day after the challenge, the concentrations of TNF-α, IFN-γ, and IL-10 in intestinal fluid and serum were determined by ELISA. The results represent three independent experiments. Results are expressed as mean ± SD. Significantly different from control mice *(*P* < 0.05).

Taking into consideration that the capacity of increasing IgA production in the gut, and stimulating macrophages and dendritic cells are amongst the beneficial effects of lactobacilli on the immune system (30, 32), we next aimed to evaluate *in vivo* the ability of *L. fermentum* UCO-979C to modulate those parameters. As shown in Figure 4, administration of the UCO-979C strain significantly increased the phagocytic activity of peritoneal macrophages while this effect was absent in the case of CRL973 strain. In order to study the activation of respiratory burst in peritoneal macrophages, we used the NBT method as described previously (30). Both, UCO-979C and CRL973 treatments were equally effective for increasing the percentage of NBT^+^ cells in the population of macrophages obtained from the peritoneal cavity (Figure 4). In addition, mice orally treated with the UCO-979C strain had significantly higher levels of intestinal IgA antibodies than control animals while *L. fermentum* CRL973 did not induce significant changes (Figure 4). It has established that the *in vivo* immunomodulatory abilities of probiotic bacteria are in part attributable to altered production of cytokines that play pivotal roles in coordinating the immune function. Then, we analyzed the concentrations of cytokines in intestinal fluid and serum obtained from lactobacilli-treated mice, to determine the local and systemic effects induced by both *L. fermentum* strains (Figure 5). No significant differences were observed between lactobacilli-treated and control mice when intestinal and serum TNF-α concentrations were analyzed. Intestinal IFN-γ protein level was augmented by both *L. fermentum* UCO-979C and CRL973 while no differences were observed for serum IFN-γ between the groups. In addition, *L. fermentum* UCO-979C significantly increased intestinal and serum IL-10 levels, an effect that was not observed for the CRL973 strain (Figure 5). We also evaluated the levels of these three cytokines: TNF-α, IFN-γ, and IL-10, in mice after the intraperitoneal challenge with LPS (Figure 6). The inflammatory stimulus increased the concentration of intestinal and serum TNF-α, and IFN-γ in all experimental groups. However, TNF-α level was significantly lower in *L. fermentum* UCO-979C-treated mice when compared with those receiving the CRL973 strain or controls. In addition, both lactobacilli augmented the production of intestinal IFN-γ after the challenge with LPS while only UCO-979C strain increased the levels of this cytokine in serum (Figure 6). LPS challenge also increased IL-10 both in intestinal fluid and serum of mice; however, the levels of this immunoregulatory cytokine were significantly higher in *L. fermentum* UCO-979C-treated mice when compared with those receiving the CRL973 strain or controls (Figure 6).

### *L. fermentum* UCO-979C Modulates Intestinal Immune Cell Populations *in vivo*

**Figure 7:**
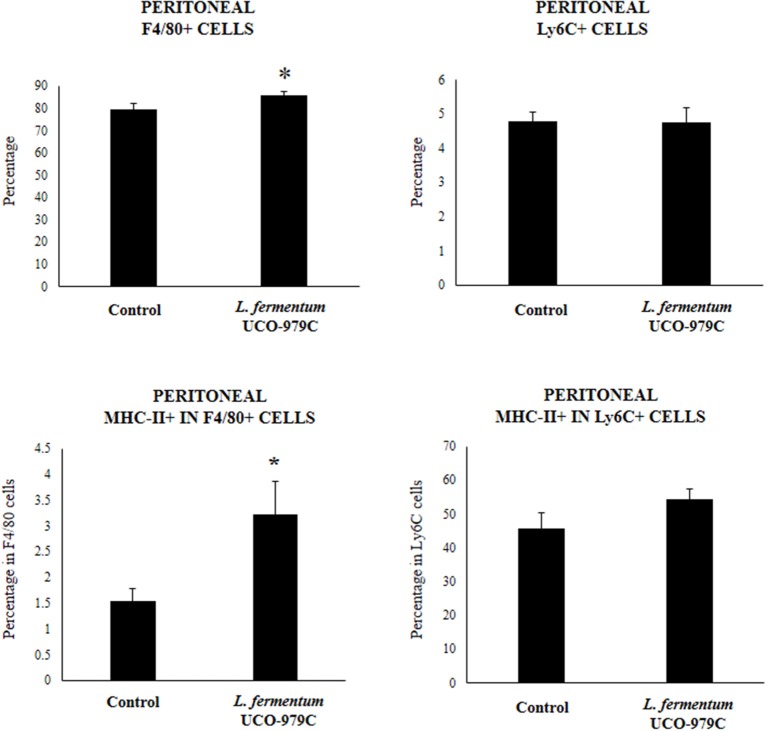
Effect of *Lactobacillus fermentum* UCO-979C on peritoneal phagocytic cells of adult immunocompetent mice. *L. fermentum* UCO-979C was administered to mice for 2 consecutive days at a dose of 10^8^ cells/mouse/day. Untreated mice were used as controls. One day after the last lactobacilli administration, peritoneal phagocytes were evaluated by flow cytometry. The results represent three independent experiments. Results are expressed as mean ± SD. Significantly different from control mice *(*P* < 0.05).

**Figure 8:**
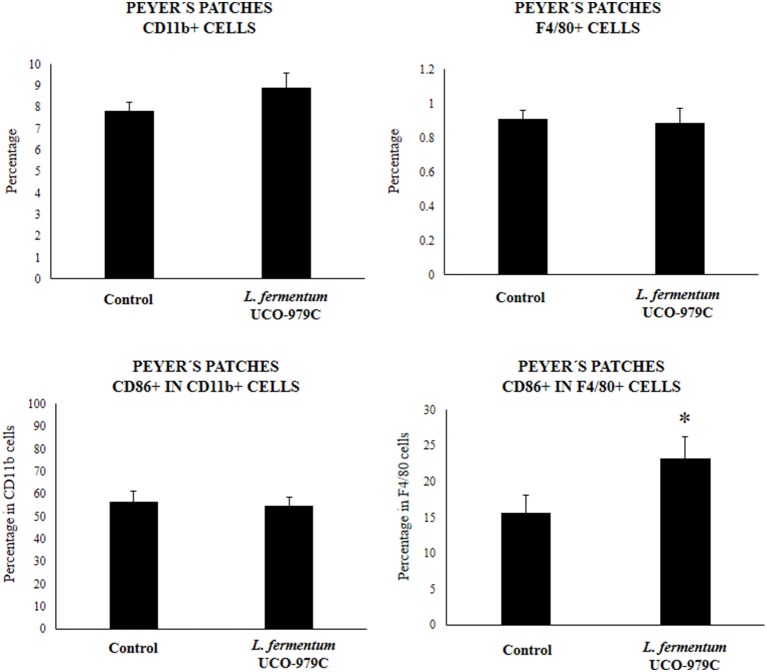
Effect of *Lactobacillus fermentum* UCO-979C on Peyer's patches phagocytic cells of adult immunocompetent mice. *L. fermentum* UCO-979C was administered to mice for 2 consecutive days at a dose of 10^8^ cells/mouse/day. Untreated mice were used as controls. One day after the last lactobacilli administration, Peyer's patches phagocytes were evaluated by flow cytometry. The results represent three independent experiments. Results are expressed as mean ± SD. Significantly different from control mice *(*P* < 0.05).

**Figure 9:**
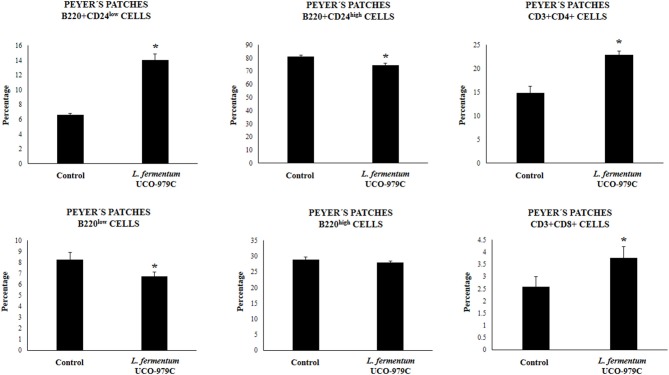
Effect of *Lactobacillus fermentum* UCO-979C on Peyer's patches lymphocytes of adult immunocompetent mice. *L. fermentum* UCO-979C was administered to mice for 2 consecutive days at a dose of 10^8^ cells/mouse/day. Untreated mice were used as controls. One day after the last lactobacilli administration, Peyer's patches T and B cells were evaluated by flow cytometry. The results represent three independent experiments. Results are expressed as mean ± SD. Significantly different from control mice *(*P* < 0.05).

We aimed to evaluate the effect of *L. fermentum* UCO-979C on peritoneal and intestinal immune cell populations in order to further characterize the immunomodulatory activity of this strain. In the peritoneal fluid, resident macrophages (F4/80^+^ cells) as well as inflammatory monocyte and neutrophils (Ly6C/Gr1^+^ cells) were studied by flow cytometry. As shown in Figure 7, the percentage of peritoneal F4/80^+^ macrophages as well as activated macrophages (F4/80^+^MCH-II^+^ cells) was increased in UCO-979C-treated mice when compared to controls, while no significant differences between the groups were observed when Ly6C/Gr1^+^ and Ly6C/Gr1^+^MHC-II^+^ cells were studied. Antigen presenting cells were also analyzed in Peyer's patches of mice (Figure 8). No significant differences were observed between UCO-979C-tretaed and control mice when CD11b^+^ (dendritic cells) or F4/80^+^ (macrophages) cells from Peyer's patches were evaluated. In addition, there were no differences between the groups in activated CD11b^+^CD86^+^ dendritic cells; however, the percentage of activated F4/80^+^CD86^+^ macrophages were significantly higher in *L. fermentum* UCO-979C-treated mice when compared with controls (Figure 8). Finally, B and T cells populations in Peyer's patches were studied (Figure 9). *L. fermentum* UCO-979C treatment improved the proportions of both CD3^+^CD4^+^ and CD3^+^CD8^+^ T cells when compared to controls. No differences between the groups were detected in the population of B220^high^ cells; however, the proportion of B220^low^ and B220^+^CD24^high^ cells (immature B cells) from Peyer's patches were significantly reduced in *L. fermentum* UCO-979C-treated mice when compared with controls (Figure 9). In addition, B220^+^CD24^low^ population (mature B cells) from Peyer's patches were higher in *L. fermentum* UCO-979C-treated mice than that of controls.

## Discussion

We previously reported that *L. fermentum* UCO-979C modulates the innate immune response in human gastric epithelial cells and macrophages, and improves protection against *H. pylori* infection (23). Here, we demonstrated for the first time that the UCO-979C strain is also capable of modulating the intestinal immune system.

The recent scientific advances in the biology of IECs have dramatically expanded our appreciation of their immunological functions. IECs establish an interconnected network with underlying immune cells and with the microbiota on their surface, and these complex interactions between intestinal cells and microorganisms significantly influence the host defense against threats from the intestinal lumen (33, 34). IECs play crucial roles in the recognition of microorganisms in both homeostatic and pathologic conditions through the expression of innate receptors including the TLRs. Signaling through TLR by pathogens in IECs initiates signaling cascades that culminates in the expression and secretion of various cytokines, chemokines, and other inflammatory factors which signal and prime underlying immune cells (33, 34). It was also reported that commensal and probiotic bacteria are recognized by IECs through innate receptors (13). Moreover, immunobiotic bacteria are able to influence TLR signaling induced by pathogens in IECs and therefore, to differentially modulate immune responses (13).

**Figure 10:**
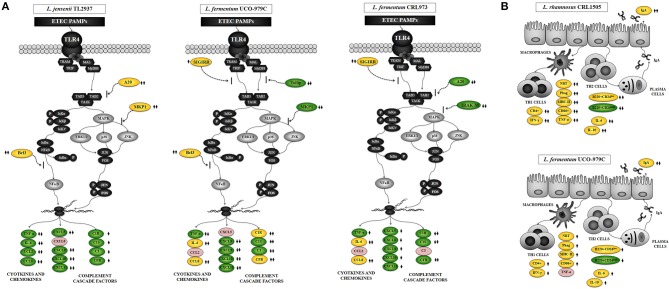
Proposed mechanisms for the immunomodulatory activity of *Lactobacillus fermentum* UCO-979C on intestinal mucosa. **(A)** Effect of *L. fermentum* UCO-979C on intestinal epithelial cells and modulation of the inflammatory response triggered by heat-stable Enterotoxigenic *Escherichia coli* (ETEC) pathogen-associated molecular patterns (PAMPs). The immunomodulatory activity of the UCO-979C strain is compared with the activity of the probiotic strain *Lactobacillus jensenii* TL2937 and with *L. fermentum* CRL973. **(B)** Effect of *L. fermentum* UCO-979C on intestinal immune cells. The immunomodulatory activity of the UCO-979C strain is compared with the activity of the probiotic strain *Lactobacillus rhamnosus* CRL1505.

We have previously used PIE cells to evaluate the effect of immunobiotic bacteria on TLR signaling induced by PAMPs. We demonstrated that stimulation of PIE cells with heat-stable ETEC PAMPs activates NF-kB and induces the phosphorylation of MAPK-ERK, MAPK-p38, and MAPK-JNK leading to the production of inflammatory cytokines (25). Later, by performing transcriptomic studies we corroborated these findings by demonstrating that activation of NF-kB and MAPK pathways in PIE cells results in an increased expression of several chemokines including CCL4, CCL5, CCL8, CCL20, CXCL2, CXCL5, CXCL9, CXCL10, CXCL11, CSF2, as well as complement and coagulation factors (27). Interestingly, we have also demonstrated that prestimulation of PIE cells with the immunobiotic strain *Lactobacillus jensenii* TL2937 differentially modulated the expression of inflammatory factors produced in response to heat-stable ETEC PAMPs challenge (25, 27) (Figure 10A). The upregulation of the negative regulators MKP-1, A20, and Bcl-3 induced by the TL2937 strain in PIE cells was found to be related to the different immunotrascriptomic response after heat-stable ETEC PAMPs challenge (25, 27). Here, we performed similar experiments in order to evaluate the immunomodulatory effects of *L. fermentum* UCO-979C in IECs. Our results showed that the prestimulation of PIE cells with the UCO-979C strain differentially modulated the expression of inflammatory factors induced by the heat-stable ETEC PAMPs challenge (Figure 10A). The changes induced by *L. fermentum* UCO-979C were distinct from those previously observed for the immunobiotic strain TL2937. While *L. jensenii* TL2937 induced a clear and remarkable anti-inflammatory effect (25, 27), *L. fermentum* UCO-979C produced a stimulant/anti-inflammatory mixed effect (Figure 10A). Though some inflammatory factors such as CXCL8, CXCL9, CXCL10, CXCL11, C1S, and C3 were significantly reduced in UCO-979C-treated PIE cells; others like IL-6, CCL8, C1R, and CFB were upregulated.

The up- and down-regulation of inflammatory factors correlated with the changes induced by *L. fermentum* UCO-979C on the expression of negative regulators of TLR4 signaling as evidenced by the augmented expression of SIGIRR and Bcl3, and the reduced expression of Tollip and MKP-1 (Figure 10A). The intestinal innate immune system needs to keep a balance in TLR activation to confer protection and avoid exaggerated inflammatory responses. Several levels of negative regulation have been described for TLR activation including the expression of membrane bound suppressors and intracellular inhibitors (13, 35). In this regard, some probiotic strains have shown to reduce TLR negative regulators and control the inflammation (36). *Lactobacillus amylovorus* DSM 16698 was reported to diminish IL-1β and IL-8 levels in IECs infected with ETEC K88 through the modulation of the negative regulators Tollip and IRAK-M (37). ETEC K88-challenged pigs exhibited a reduced inflammatory response after *L. acidophilus* administration and this effect was associated with an increased expression of splenic Tollip, IRAK-M, A20, and Bcl-3 (38). In addition, *L. plantarum* CGMCC1258 increased SIGIRR, Bcl3, and MKP-1 gene expressions in porcine IECs challenged with ETEC K88 ameliorating the production of IL-8 and TNF-α (39).

The stimulating/anti-inflammatory mixed effect of *L. fermentum* UCO-979C in PIE cells contrast with our previous findings in AGS cells (23). We demonstrated that *L. fermentum* UCO-979C significantly diminished the production of IL-8, TNF-α, IL-1β, IL-6, and MCP-1 in AGS cells challenged with *H. pylori*. Of interest, in addition to its capacity to reduce the production of pro-inflammatory factors we observed that *L. fermentum* UCO-979C was also capable to improve the production of TGF-β in *H. pylori*-infected AGS cells. These findings indicate that it is of great importance to characterize the immunomodulatory properties of the probiotic strains in different cellular models since it is not possible to extrapolate their effect in one mucosal tissue to another.

Several studies have reported the beneficial effects of immunobiotics on intestinal health and those studies have shown that the most remarkable effect of immunobiotics on intestinal cytokine dynamics is the increase of TNF-α, IFN-γ, and the regulatory cytokine IL-10 (30, 40). We have shown consistently that the oral administration of immunobiotic strains including *L. casei* CRL431, *L. plantarum* CRL1506, and *L. rhamnosus* CRL1505 improves the production of TNF-α, IFN-γ, and IL-10 in the gut (30, 31, 41). Moreover, we demonstrated that oral administration of the immunostimulatory strain *L. rhamnosus* CRL1505 to mice improved the activation of intestinal and peritoneal macrophages as well as Peyer's patches CD4^+^IFN-γ^+^ T cells (30, 31, 41). In the present study, the oral administration of *L. fermentum* UCO-979C significantly improved the production of IFN-γ, and IL-10 but not the TNF-α. The UCO-979C strain stimulated intestinal and peritoneal macrophages and improved Peyer's patches CD4^+^ T cells, although the effects were less pronounced than those previously observed for the CRL1505 strain (Figure 10B). These results indicate that *L. fermentum* UCO-979C would be capable of stimulating CD4^+^ T cells in the gut, increasing IFN-γ production and consequently stimulating the macrophages. In fact, peritoneal macrophages of UCO-979C-treated mice had improved levels of parameters that are involved in several fundamental steps of the phagocytic process including attachment to surface and internalization of *S. boulardii* as well as their microbicidal activity through oxidative burst. We previously reported that *L. fermentum* UCO-979C modulated cytokine production in THP-1 macrophages challenged either with *H. pylori* or LPS (23). The UCO-979C strain was able to reduce the production of TNF-α, and to improve IFN-γ levels in challenged THP-1 macrophages. In addition, we demonstrated that *L. fermentum* UCO-979C increased the production of IL-10 in THP-1 macrophages challenged with *H. pylori* (23). Our previous results and those obtained in this work therefore suggest that the UCO-979C strain could exert an immunomodulatory effect on macrophages acting directly on them or indirectly through the cytokines produced by IECs or other immune cells.

Another effect that has been consistently described for probiotics on the intestinal immune system is their ability to improve secretory IgA production, which is supported in most cases by an improved production of factors released by IECs. Cytokines produced by IECs such as IL-6 are capable of promoting the switch from IgM to IgA expression in B cells (31, 40). It is considered that approximately 80% of antibody-secreting plasma cells in the human body are located in the gut. Intestinal antibody-secreting plasma cells produce secretory IgA that plays an important protective role against pathogens and toxins through a variety of non-inflammatory activities that increase their clearance, and prevent their access to the intestinal epithelium (42). Dimeric IgA also neutralize endocytosed LPS in intestinal epithelium, preventing NF-κB activation (43). IgA-producing B cells can be generated by both T cell-dependent and -independent processes (44). T cell-dependent responses usually occur in germinal centers in lymphoid tissues such as the Peyer's patches and mesenteric lymph nodes. In such structures, B cells undergo several rounds of activation and maturation that are supported by follicular T cells that express co-stimulatory molecules and cytokines. In T-independent responses, which occur outside germinal centers, B cells are activated by IECs and innate immune cells to produce polyreactive IgA (45). The T-independent IgA production is induced by and influences the composition of indigenous members of the microbiota (42). In addition, improvement of T-independent IgA induction supported by TGF-β, IL-4, IL-2, IL-6, and IL-10 was also demonstrated for immunobiotic strains including *L. casei* CRL431 (40), *L. rhamnosus* GG (46), and *L. rhamnosus* CRL1505 (31). In this work, we have observed that the oral administration of the *L. fermentum* UCO-979C improves intestinal IL-6, reduces immature B220^+^CD24^high^ B cells from Peyer's patches, enhances mature B B220^+^CD24^low^ cells and significantly increases intestinal IgA, although these effects were less pronounced than those observed for the CRL1505 strain (Figure 10B).

Interestingly, *L. fermentum* CRL973 showed a modest immunomodulatory effect *in vitro* (Figure 10A). The CRL973 strain increased the expression of SIGIRR and reduced the expression of the negative regulators A20 and IRAK-M in PIE cells after heat-stable ETEC PAMPs challenge. However, the *in vivo* studies in mice demonstrated that the CRL973 strain was not able to exert an immunomodulatory effect since no improvement of intestinal cytokines, IgA production, or activation of peritoneal macrophages was observed in the CRL973-treated mice. These findings are of importance since they confirm the general knowledge that the immunobiotic properties are dependent on each specific strain. Moreover, results of this study open up an interesting possibility for future research since cellular, molecular, and genomic comparative studies between both UCO-979C and CRL973 strains could help to understand the immunological mechanisms involved in the beneficial effects of *L. fermentum* UCO-979.

We have demonstrated that *L. fermentum* UCO-979C is able to differentially modulate the cytokine response of human gastric epithelial cells and macrophages, and to improve protection against *H. pylori* infection *in vitro* (23). The UCO-979C strain is also capable to modulate the immune response of IECs triggered by heat-stable ETEC PAMPs challenge. Notably, we demonstrated here for the first time that *L. fermentum* UCO-979C is able to exert its immunomodulatory effect in the intestinal mucosa *in vivo*. Therefore, *L. fermentum* UCO-979C has several characteristics for making it an excellent candidate for the development of immunobiotic functional foods to prevent infections by gastric and intestinal pathogens. The *in vivo* evaluation of the ability of the UCO-979C strain to beneficially influence the immune response and improve protection against *H. pylori* and other intestinal pathogenic Gram-negative bacteria is an interesting point for further research.

## Data Availability

The raw data supporting the conclusions of this manuscript will be made available by the authors, without undue reservation, to any qualified researcher.

## Ethics Statement

This study was carried out in strict accordance with the recommendations in the Guide for the Care and Use of Laboratory Animals of the Guidelines for Animal Experimentation of CERELA. The CERELA Institutional Animal Care and Use Committee prospectively approved this research under the protocol BIOT-CRL-17.

## Author Contributions

JV and HK designed the study. VG-C, RK, YI, MT, and MI did the *in vitro* experiments. VG-C, PC, and SS did the *in vivo* experiments. VG-C, JV, HK, and HT provided financial support. VG-C, JV, HK, AG-C, and SA contributed to data analysis and results interpretation. VG-C, JV, HK, and MI wrote the manuscript. HK, JV, and AG-C approved the final version of manuscript.

### Conflict of Interest Statement

The authors declare that the research was conducted in the absence of any commercial or financial relationships that could be construed as a potential conflict of interest.

## References

[1] Dicks LMT Geldenhuys J Mikkelsen LS Brandsborg E Marcotte H. Our gut microbiota: a long walk to homeostasis. Benef Microbes. (2018) 9:3–20. 10.3920/BM2017.0066 29022388

[2] Sánchez B Delgado S Blanco-Míguez A Lourenço A Gueimonde M Margolles A Probiotics, gut microbiota, and their influence on host health and disease. Mol Nutr Food Res. (2017) 6:1–15. 10.1002/mnfr.201600240 27500859

[3] Hooper LV Littman DR Macpherson AJ. Interactions between the microbiota and the immune system. Science. (2012) 336:1268–73. 10.1126/science.1223490 22674334 PMC4420145

[4] Hevia A Delgado S Sánchez B Margolles A. Molecular players involved in the interaction between beneficial bacteria and the immune system. Front Microbiol. (2015) 6:1285 10.3389/fmicb.2015.01285 26635753 PMC4649051

[5] Neville BA Forster SC Lawley TD. Commensal Koch's postulates: establishing causation in human microbiota research. Curr Opin Microbiol. (2018) 42:47–52. 10.1016/j.mib.2017.10.001 29112885

[6] Highlander SK. High throughput sequencing methods for microbiome profiling: application to food animal systems. Anim Health Res Rev. (2012) 13:40–53. 10.1017/S1466252312000126 22853944

[7] Wang B Yao M Lv L Ling Z Li L The human microbiota in health and disease. Engineering. (2017) 3:71–82. 10.1016/J.ENG.2017.01.008

[8] Perdigón G Fuller R Raya R Lactic acid bacteria and their effect on the immune system further reading. Curr Issues Intest Microbiol. (2001) 2:27–42. 11709854

[9] Clancy R. Immunobiotics and the probiotic evolution. FEMS Immunol Med Microbiol. (2003) 38:9–12. 10.1016/S0928-8244(03)00147-0 12900049

[10] Kitazawa H Villena J Alvarez S Probiotics: Immunobiotics and Immunogenics. CRC Press (2013). Available online at: https://books.google.com/books?id=FJ3NBQAAQBAJ&pgis=1

[11] Wu R Jeffrey M Johnson-Henry K Green-Johnson J Sherman P Impact of prebiotics, probiotics and gut derived metabolites on host immunity. LymphoSign J. (2016) 4:1–24. 10.14785/lymphosign-2016-0012

[12] Lebeer S Bron PA Marco ML Van Pijkeren JP O'Connell Motherway M Hill C. Identification of probiotic effector molecules: present state and future perspectives. Curr Opin Biotechnol. (2018) 49:217–23. 10.1016/j.copbio.2017.10.007 29153882

[13] Villena J Kitazawa H. Modulation of intestinal TLR4-inflammatory signaling pathways by probiotic microorganisms: lessons learned from *Lactobacillus jensenii* TL2937. Front Immunol. (2014) 4:512 10.3389/fimmu.2013.00512 24459463 PMC3890654

[14] Ren C Zhang Q De Haan BJ Zhang H Faas MM De Vos P. Identification of TLR2/TLR6 signalling lactic acid bacteria for supporting immune regulation. Sci Rep. (2016) 6:34561 10.1038/srep34561 27708357 PMC5052581

[15] Barberi C Campana S De Pasquale C Rabbani Khorasgani M Ferlazzo G Bonaccorsi I. T cell polarizing properties of probiotic bacteria. Immunol Lett. (2015) 168:337–42. 10.1016/j.imlet.2015.11.005 26554608

[16] Bron PA Kleerebezem M Brummer RJ Cani PD Mercenier A MacDonald TT. Can probiotics modulate human disease by impacting intestinal barrier function? Br J Nutr. (2017) 117:93–107. 10.1017/S0007114516004037 28102115 PMC5297585

[17] Lesbros-Pantoflickova D Corthésy-Theulaz I Blum AL. *Helicobacter pylori* and probiotics. J Nutr. (2007) 137:812S−8S. 10.1093/jn/137.3.812S 17311980

[18] Goderska K Agudo Pena S Alarcon T *Helicobacter pylori* treatment: antibiotics or probiotics. Appl Microbiol Biotechnol. (2017) 102:1–7. 10.1007/s00253-017-8535-7 29075827 PMC5748437

[19] Homan M Orel R. Are probiotics useful in *Helicobacter pylori* eradication? World J Gastroenterol. (2015) 21:10644–53. 10.3748/wjg.v21.i37.10644 26457024 PMC4588086

[20] Garcia CA Henriquez AP Retamal RC Pineda CS Delgado Sch C Gonzalez CC Probiotic properties of *Lactobacillus* spp isolated from gastric biopsies of *Helicobacter pylori* infected and non-infected individuals. Rev Med Chil. (2009) 137:369–76. 10.4067/S0034-98872009000300007 19621178

[21] García A Navarro K Sanhueza E Pineda S Pastene E Quezada M Characterization of *Lactobacillus fermentum* UCO-979C, a probiotic strain with a potent anti-*Helicobacter pylori* activity. Electron J Biotechnol. (2017) 25:75–83. 10.1016/j.ejbt.2016.11.008

[22] Merino JS García A Pastene E Salas A Saez K González CL. *Lactobacillus fermentum* UCO-979C strongly inhibited *Helicobacter pylori* SS1 in *Meriones unguiculatus*. Benef Microbes. (2018) 9:625–7. 10.3920/BM2017.0160 29633633

[23] Garcia-Castillo V Zelaya H Ilabaca A Espinoza-Monje M Komatsu R Albarracin L. *Lactobacillus fermentum* UCO-979C beneficially modulates the innate immune response triggered by *Helicobacter pylori* infection *in vitro*. Benef Microbes. (2018) 9:829–41. 10.3920/BM2018.0019 29798705

[24] Moue M Tohno M Shimazu T Kido T Aso H Saito T. Toll-like receptor 4 and cytokine expression involved in functional immune response in an originally established porcine intestinal epitheliocyte cell line. Biochim Biophys Acta Gen Subj. (2008) 1780:134–44. 10.1016/j.bbagen.2007.11.006 18082146

[25] Shimazu T Villena J Tohno M Fujie H Hosoya S Shimosato T. Immunobiotic *Lactobacillus jensenii* elicits anti-inflammatory activity in porcine intestinal epithelial cells by modulating negative regulators of the toll-like receptor signaling pathway. Infect Immun. (2012) 80:276–88. 10.1128/IAI.05729-11 22083706 PMC3255675

[26] Tomosada Y Villena J Murata K Chiba E Shimazu T Aso H. Immunoregulatory effect of bifidobacteria strains in porcine intestinal epithelial cells through modulation of ubiquitin-editing enzyme A20 expression. PLoS ONE. (2013) 8:e59259 10.1371/journal.pone.0059259 23555642 PMC3608626

[27] Kobayashi H Albarracin L Sato N Kanmani P Kober AKMH Ikeda-Ohtsubo W. Modulation of porcine intestinal epitheliocytes immunetranscriptome response by *Lactobacillus jensenii* TL2937. Benef Microbes. (2016) 7:769–82. 10.3920/BM2016.0095 27824278

[28] Nygard AB Jorgensen CB Cirera S Fredholm M. Selection of reference genes for gene expression studies in pig tissues using SYBR green qPCR. BMC Mol Biol. (2007) 8:67. 10.1186/1471-2199-8-67 17697375 PMC2000887

[29] Ray A Dittel BN Isolation of mouse peritoneal cavity cells. J Vis Exp. (2010) 35:1488 10.3791/1488 PMC3152216 20110936

[30] Marranzino G Villena J Salva S Alvarez S. Stimulation of macrophages by immunobiotic *Lactobacillus* strains: influence beyond the intestinal tract. Microbiol Immunol. (2012) 56:771–81. 10.1111/j.1348-0421.2012.00495.x 22846065

[31] Salva S Villena J Alvarez S. Immunomodulatory activity of *Lactobacillus rhamnosus* strains isolated from goat milk: impact on intestinal and respiratory infections. Int J Food Microbiol. (2010) 141:82–9. 10.1016/j.ijfoodmicro.2010.03.013 20395002

[32] Tsukida K Takahashi T Iida H Kanmani P Suda Y Nochi T. Immunoregulatory effects triggered by immunobiotic *Lactobacillus jensenii* TL2937 strain involve efficient phagocytosis in porcine antigen presenting cells. BMC Immunol. (2016) 17:1–12. 10.1186/s12865-016-0160-1 27342653 PMC4921007

[33] Abreu MT. Toll-like receptor signalling in the intestinal epithelium: how bacterial recognition shapes intestinal function. Nat Rev Immunol. (2010) 10:131–44. 10.1038/nri2707 20098461

[34] Allaire JM Crowley SM Law HT Chang SY Ko HJ Vallance BA. The intestinal epithelium: central coordinator of mucosal immunity. Trends Immunol. (2018) 39:677–96. 10.1016/j.it.2018.04.002 29716793

[35] Liew FY Xu D Brint EK O'Neill LAJ. Negative regulation of toll-like receptor-mediated immune responses. Nat Rev Immunol. (2005) 5:446–58. 10.1038/nri1630 15928677

[36] Llewellyn A Foey A. Probiotic modulation of innate cell pathogen sensing and signaling events. Nutrients. (2017) 9:1–21. 10.3390/nu9101156 29065562 PMC5691772

[37] Finamore A Roselli M Imbinto A Seeboth J Oswald IP Mengheri E. *Lactobacillus amylovorus* inhibits the TLR4 inflammatory signaling triggered by enterotoxigenic *Escherichia coli* via modulation of the negative regulators and involvement of TLR2 in intestinal caco-2 cells and pig explants. PLoS ONE. (2014) 9:e94891. 10.1371/journal.pone.0094891 24733511 PMC3986366

[38] Li H Zhang L Chen L Zhu Q Wang W Qiao J. *Lactobacillus acidophilus* alleviates the inflammatory response to enterotoxigenic *Escherichia coli* K88 via inhibition of the NF-κB and p38 mitogen-activated protein kinase signaling pathways in piglets. BMC Microbiol. (2016) 16:273. 10.1186/s12866-016-0862-9 27832756 PMC5105324

[39] Wu Y Zhu C Chen Z Chen Z Zhang W Ma X. Protective effects of *Lactobacillus plantarum* on epithelial barrier disruption causedby enterotoxigenic *Escherichia coli* in intestinal porcine epithelial cells. Vet Immunol Immunopathol. (2016) 172:55–63. 10.1016/j.vetimm.2016.03.005 27032504

[40] Maldonado C De Moreno De Leblanc A Vinderola G Bibas Bonet ME Perdigón G Proposed model: mechanisms of immunomodulation induced by probiotic bacteria. Clin Vaccine Immunol. (2007) 14:485–92. 10.1128/CVI.00406-06 17360855 PMC1865623

[41] Villena J Chiba E Tomosada Y Salva S Marranzino G Kitazawa H. Orally administered *Lactobacillus rhamnosus* modulates the respiratory immune response triggered by the viral pathogen-associated molecular pattern poly(I:C). BMC Immunol. (2012) 13:53. 10.1186/1471-2172-13-53 22989047 PMC3460727

[42] Jahnsen FL Bækkevold ES Hov JR Landsverk OJ. Do long-lived plasma cells maintain a healthy microbiota in the gut? Trends Immunol. (2018) 39:196–208. 10.1016/j.it.2017.10.006 29162322

[43] Cario E Podolsky DK. Intestinal epithelial tollerance versus intollerance of commensals. Mol Immunol. (2005) 42:887–93. 10.1016/j.molimm.2004.12.002 15829278

[44] Pabst O. New concepts in the generation and functions of IgA. Nat Rev Immunol. (2012) 12:821–32. 10.1038/nri3322 23103985

[45] Stephens WZ Round JL. IgA targets the troublemakers. Cell Host Microbe. (2014) 16:265–7. 10.1016/j.chom.2014.08.012 25211066

[46] Wang Y Liu L Moore DJ Shen X Peek RM Acra SA. An LGG-derived protein promotes IgA production through upregulation of APRIL expression in intestinal epithelial cells. Mucosal Immunol. (2017) 10:373–84. 10.1038/mi.2016.57 27353252 PMC5199635

